# Global stakeholder perspectives of home birth: a systematic scoping review

**DOI:** 10.1186/s13643-021-01837-9

**Published:** 2021-11-02

**Authors:** Ginny Brunton, Samira Wahab, Hassan Sheikh, Beth Murray Davis

**Affiliations:** 1 Faculty of Health Sciences, Ontario Tech University, Oshawa, ON Canada; 2 McMaster Midwifery Research Centre, Department of Obstetrics and Gynecology, Faculty of Health Sciences, McMaster University, Hamilton, ON Canada

**Keywords:** Home birth, Systematic review, Stakeholders, Health policy, Perspectives, Qualitative research

## Abstract

Home birth is experienced by people very differently worldwide. These experiences likely differ by the type of stakeholder involved (women, their support persons, birth attendants, policy-makers), the experience itself (low-risk birth, transfer to hospital, previous deliveries), and by the health system within which home birth occurs (e.g., high-resource versus low- and middle-resource countries). Research evidence of stakeholders’ perspectives of home birth could usefully inform personal and policy decisions about choosing and providing home birth, but the current literature is fragmented and its breadth is not fully understood.

We conducted a systematic scoping review to understand how the research literature on stakeholders’ perspectives of home birth is characterized in terms of populations, settings and identified issues, and what potential gaps exist in the research evidence. A range of electronic, web-based and key informant sources of evidence were searched. Located references were assessed, data extracted, and descriptively analyzed using robust methods.

Our analysis included 460 full reports. Findings from 210 reports of studies in high-resource countries suggested that research with fathers and same-sex partners, midwives, and vulnerable populations and perspectives of freebirth and transfer to hospital could be synthesized. Gaps in primary research exist with respect to family members, policy makers, and those living in rural and remote locations. A further 250 reports of studies in low- and middle-resource countries suggested evidence for syntheses related to fathers and other family members, policy makers, and other health care providers and examination of issues related to emergency transfer to hospital, rural and remote home birth, and those who birth out of hospital, often at home, despite receiving antenatal care intended to increase healthcare-seeking behavior. Gaps in primary research suggest an examination is needed of perspectives in countries with higher maternal mortality and among first-time mothers and young mothers.

Our scoping review identified a considerable body of research evidence on stakeholder perspectives of home birth. These could inform the complex factors influencing personal decisions and health system planning around home birth in both high- and low- and middle-resource countries. Future primary research is warranted on specific stakeholders worldwide and with vulnerable populations in areas of high maternal mortality.

## Background

### Rationale

Childbirth is an important event for a woman and her family [1–3]. Birth takes place in many settings worldwide including hospitals, home, freestanding- and alongside-midwifery units, and community maternity and primary health centers [4–7]. Home births are experienced by women in both high- and low-resource countries [8]; however, the circumstances surrounding these home births are very different.

Between 1 and 16% of childbearing people in high-resource countries (HRCs) choose to give birth at home, where midwifery services are well-integrated into the health system model of care [9–12]. Here, women are supported to make informed choices about their maternity care, including where to give birth [4, 5, 10, 13, 14]. For women at low risk of complications, this integration of health system support for home birth in high-resource countries has been associated with maternal and newborn outcomes similar to those of women experiencing hospital delivery [15, 16]. However, in low- and middle-resource countries (LMRCs), birth at home occurs much more frequently and may be associated with high mortality, although the number of facility-based births is increasing [17, 18]. Up until 2016, an estimated 0.3 to 90.6% of all births take place outside of healthcare institutions in low- and middle-resource countries worldwide [19]. In 2017, an estimated 810 women worldwide died daily from preventable complications of pregnancy and childbirth, with 94% of these occurring in low-resource settings [20]. In low-resource settings, the provision of safe home birth is complicated by multiple factors. These include a lack of skilled birth attendants [21, 22]; access to, and quality of, care in obstetric facilities in the event of complications [6, 7]; pregnant women’s knowledge of complications [23, 24]; their cultural beliefs [25, 26]; and societal norms related to women’s autonomy [27].

Within this myriad of personal, societal, and health system influences, women decide whether to birth at home, their partners decide whether and how to support them, and practitioners and policy makers plan, deliver, and evaluate home birth services. Evidence from research examining the experiences of key stakeholders, including women, partners, family members, health care practitioners, policy and decision-makers, could usefully inform personal and policy decisions regarding home birth. Different stakeholders’ perspectives can provide an important source of evidence underpinning quality of care in both hospital facilities and planned home births [28]. Listening to diverse women’s experiences of birth, across different countries and under varied circumstances, can also address gender equity issues in health [29].

A large amount of qualitative research on women’s experiences of home birth exists and may be useful, however, this evidence comes from diverse populations. Several previous systematic reviews have examined the perspectives of women, midwives and nurses. The majority of these focus on women’s views in high-resource countries [30–39]. Of these, six are now outdated; and the remaining current reviews focus on perspectives in the United Kingdom only [31, 39] or are systematic review protocols [34, 35]. Fewer systematic reviews focus on practitioners’ perspectives of home birth: three systematic reviews examined the perspectives of midwives and nurses, and of these, two conducted with stakeholders in HRCs are no longer current [40, 41]. The third examines midwives’ perspectives of home birth in LMRCs [42]. No systematic reviews were located that sought the perspectives of other key stakeholders in high- or low-resource settings, including those of fathers, other health care providers or policy decision-makers.

The primary research exploring perspectives other than women or practitioners is disparate and may provide conflicting findings in countries where home birth is, and is not, well integrated [43–49]. These primary studies have also highlighted a range of topic areas, including decision-making and satisfaction, the characteristics and risk status of those who choose home birth, and the influence of geographic location on birthplace [50–59].

To facilitate evidence-informed decision-making among women, their support persons, health care professionals, and those setting clinical and policy standards, there is a need to understand the body of research examining the experiences and perspectives of home birth as voiced by those who are involved in home birth decisions. This involves first understanding the breadth of evidence that has been conducted and the types of participants, settings and contexts in which home birth research has been conducted.

By mapping or scoping the breadth of research on people’s perspectives of home birth, our aim is to understand the range of debates on the value and place of home birth in different communities and among different populations. By mapping or scoping the breadth of research on people’s perspectives of home birth, our aim is to understand the range of debates on the value and place of home birth in different communities and among different populations. Collating this research literature will identify the breadth of research on this topic, examining determinants such as ethnicity, socioeconomic status, disability, sexual/gender orientation, migration status, age, geography, and with health system factors which interact with issues of sex or gender and influence birth processes and outcomes [60, 61]. Such understanding could help women decide where to give birth, allow clinicians to provide individualized, evidence-informed care that enlightens and supports choice, and help policy decision-makers assess the fit between the research evidence, service user needs and values, and the availability of birth place options [62]. To foster these decisions, we conducted a scoping review to identify the breadth and nature of the evidence worldwide.

### Objectives

In order to prioritize the populations and topics where home birth research exists and could be synthesized usefully for evidence-informed policy, practice and personal decisions, the research landscape must be understood [63]. A scoping review is best suited to this purpose. Scoping reviews utilize systematic review methods and map the breadth of research undertaken on home birth, its key characteristics, and any evidence gaps where future primary research is needed [63, 64]. Two research questions were addressed:

1. How is the research literature on stakeholder perspectives, opinions, and views of planned home birth characterized in terms of populations, settings, and identified issues?
2. Where are there gaps in the research evidence on stakeholder perspectives of planned home birth?

A review protocol was published on the McMaster Midwifery Research Centre’s website in March 2020 [65].

## Methods

### Stakeholder engagement

As part of a larger CIHR-funded knowledge planning and dissemination grant (CIHR #162186), we consulted an advisory group comprised of midwifery researchers, obstetric, family practice and midwifery practitioners, and a member of the public with maternity policy expertise. Advisory group members were consulted twice: first to advise on review methods and identify potentially relevant key research and second to consider and interpret emerging findings.

### Search strategy

Searches were updated from our previous systematic review of home birth [15, 16] and supplemented using a range of medical and social databases, including EMBASE, MEDLINE, CINAHL, AMED, ASSIA, ProQuest Thesis Dissertations, and Cochrane Library. In addition, key websites (e.g., World Health Organization and International Congress of Midwives) and journal hand searching (e.g., Canadian Journal of Midwifery Research, Birth, British Midwifery Journal, Midwifery) were conducted. Searches were conducted from January 2010 to April 2021. Search terms incorporated subject headings and free-text terms for home birth or home delivery, translated from MEDLINE out to other databases. A search strategy and resultant outputs for all databases is provided in Appendix.

### Eligibility criteria

All identified references were screened hierarchically and included in the review if they:

- Were an empirical study (containing a description of an identified sample, data collection and analysis);
- Concerned women/clients who planned to or did give birth at home;
- Aimed to elicit views, perspectives, opinions or experiences of any stakeholders (including women/clients, partners, health care providers, policy and decision-makers);
- Were published in English or are translatable; and
- Were published from 2010 onward.

We excluded narrative descriptive birth stories that described events or outcomes, but any research including rationales, reasons, motivation, values, perspectives, and decision-making as part of the descriptive were included. As English language is the research team’s primary language, this was the minimum required for adequate eligibility screening. However, studies in other languages were marked for possible future research. To ensure that the included research reflected current professional guidance and stakeholder perspectives on home birth, a date limit of 2010 was used. This reflected either the date since data were collected or when the reference was published. Studies were also excluded that focused solely on hospital birth or alternative birth center settings.

### Data extraction

Data from included studies were extracted using previously developed modified coding frameworks that were informed by the advisory group [31, 65]. Extracted data included study aims, geographic location, type of participant (women/clients, partner, clinician, policy maker), or parity. High- and low- and middle-resource countries were defined as per current World Bank definitions [8]. Emerging findings were shared during consultations with advisory group members. This helped the research team to interpret the findings, evaluate our methods, and determine future research and dissemination collaborations to support evidence-informed decision-making.

### Data analysis

To establish the breadth of research conducted on home birth, extracted study characteristics were descriptively analyzed and summarized in tabular format. Descriptive frequencies of all study characteristics (e.g., country or origin, year of publication, type of stakeholder, topic under study) were calculated using Excel. Assessing risk of bias or critical appraisal of studies was not undertaken, as this stage is not appropriate for a scoping review where the aims are to understand the nature and breadth of research in an area of inquiry [66, 67]. Similarly, assessment of both meta-biases and confidence in cumulative evidence are not appropriate for a scoping review [66, 68]. Findings from the scoping review are reported separately for high-resource countries (HRC) and low- and middle-resource countries (LMRC). This is due to differences in health systems provision of integrated home birth services, which may influence both stakeholders’ perspectives as well as the nature of the research conducted [9, 69].

### Quality assurance

EPPI-Reviewer software was utilized for review processes. The protocol and final report were developed as per PRISMA(-P) reporting guidelines [70, 71]. For screening and data extraction, two researchers worked independently, establishing agreement on a common sub-set of studies with disagreements resolved by a third researcher.

## Results

### Protocol alterations

As a scoping review, registration was not possible in PROSPERO’s systematic review register. Instead, the protocol was made publicly available on McMaster University’s Midwifery Research Centre website [67]. Two alterations in the protocol were made at the request of and confirmation by our Advisory Group. First, very few studies reported whether the women planning or experiencing home birth were considered to be of low risk. Therefore, all studies of women who planned or gave birth at home, regardless of risk status, were included in the scoping review. Second, a high number of studies conducted in LMRCs focused on reasons for home birth despite access to health facilities. This was considered by the Advisory Group to be an important need to understand; thus these studies were also included for analysis.

### Overall findings

**Fig. 1:**
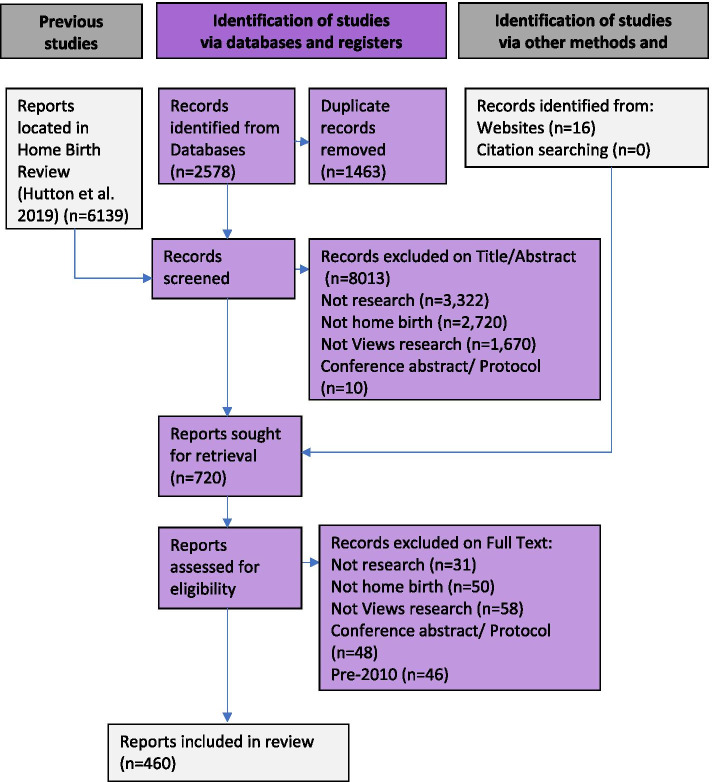
PRISMA diagram

Searching identified 10,196 references. After duplicate removal and screening on title and abstract, a total of 720 references were retrieved for assessment based on the full report. Of these, 460 met the eligibility criteria and were included in the scoping review analysis. Most studies were excluded at title/abstract or full text screening stages because they were not empirical research (*n* = 3353), were not focused on home birth (*n* = 2770), or were not research on people’s perspectives (*n* = 1728). The flow of studies through the review is provided in Fig. 1.

Stakeholder perspectives of home birth have been studied globally across 73 different countries. A range of focus group, interviews and qualitative survey designs were employed. Studies were distributed fairly equally between HRCs and LMRCs [8], with 54% conducted in LMRCs compared to 46% in HRCs.

### Home birth in high-resource countries

**Fig. 2:**
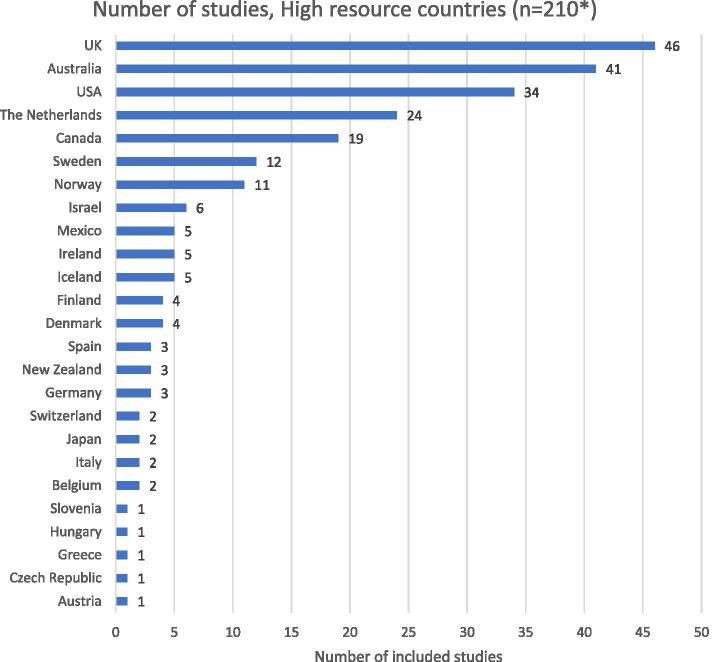
High-resource countries conducting stakeholder research

A total of 210 studies examining stakeholder research on home birth were located within 25 HRCs, as illustrated in Fig. 2.

Research examining stakeholders’ views of home birth was conducted most often in the UK (22% of 210 HRC-based studies), followed by Australia (20%), the USA (16%), the Netherlands (11%), and Canada (9%). It should be noted that some studies sampled from more than one country, hence numbers add up to the overall totals for HRCs reported above. Several HRCs were not represented, including Chile and Colombia, South Korea, and several countries within Europe (i.e., Estonia, Latvia, Lithuania, France, Luxembourg, Hungary, Poland, Portugal, Slovak Republic, and Turkey).

#### Populations studied in high-resource countries

**Fig. 3:**
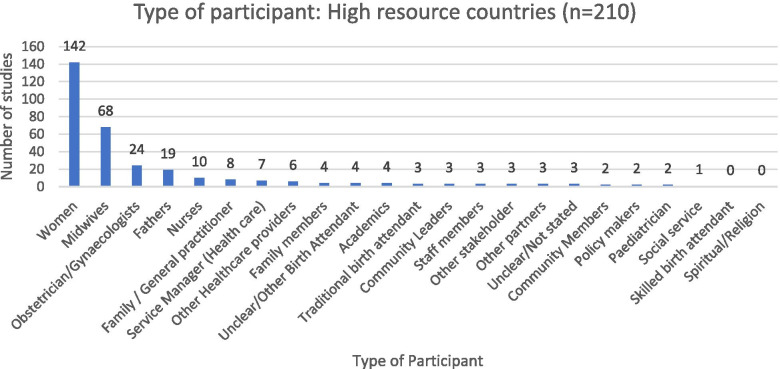
High-resource countries: type of participant

The experiences of a wide range of clients, support persons, and health care practitioners were sought across high-resource countries, as illustrated in Fig. 3.

Women’s experiences were most often reported (142 studies, 68% of 210 HRCs), although the perspectives of fathers (9%) and family members (2%) were also included in some studies. Midwives were the most frequently researched healthcare providers (32%), followed by obstetricians (11%), nurses (5%) and family practitioners (4%). Pediatricians (1%) and other health care providers (including doulas and traditional birth attendants) were studied less often (3%). The views of service managers (4%) and staff members (2%) were studied infrequently. The perspectives of community leaders (*n* = 3) and policy makers (*n* = 2) regarding home birth were rarely studied.

#### Home birth issues studied in high-resource countries

**Fig. 4:**
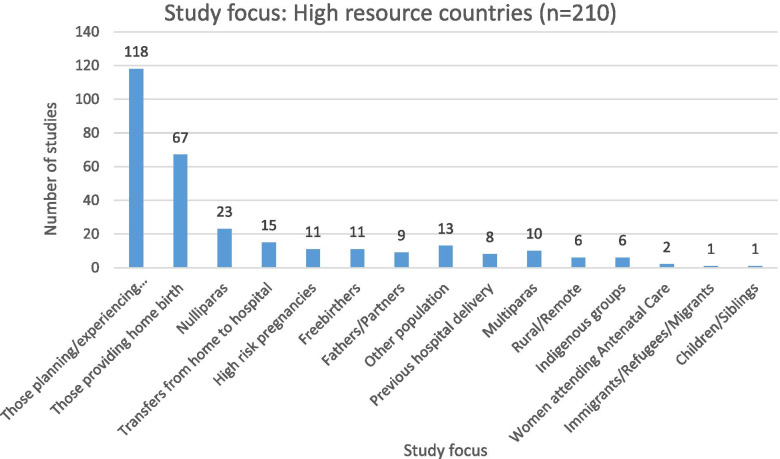
High-resource countries: study focus

A range of issues related to home birth were studied within high-resource countries, as shown in Fig. 4.

Within HRCs, home birth studies focused most often on the perspectives of those who were planning or had experienced home birth (118 studies, 56% of 210 studies from HRCs). Another 67 studies (32%) focused on understanding home birth from the perspectives of those providing home birth services.

The perspectives of different types of clients were also the main focus of a group of studies. Perspectives of home birth among women experiencing their first pregnancy were reported in 23 studies within HRCs (11%), with only 5% asking multiparous women. The experiences of rural and remote participants (3%), Indigenous groups (3%), and immigrants (one study) were less often reported.

Some studies also explored purposely the views of partners and family members: the perspectives of fathers or partners were the specific focus in nine studies (4%). Only one study sought the experiences of children/siblings who were present during home births.

Perspectives on risk in home birth were also a focus. A total of 11 studies (5%) considered the experiences of those who chose to freebirth. The experiences of home birth for those with high-risk pregnancy were reported in eleven studies (5%) and of women who had a previous hospital delivery in eight studies (4%). The perspectives of those who experienced transfer from home to hospital during birth were reported in 15 of the included HRC studies (7%).

### Home birth in low- and middle-resource countries

**Fig. 5:**
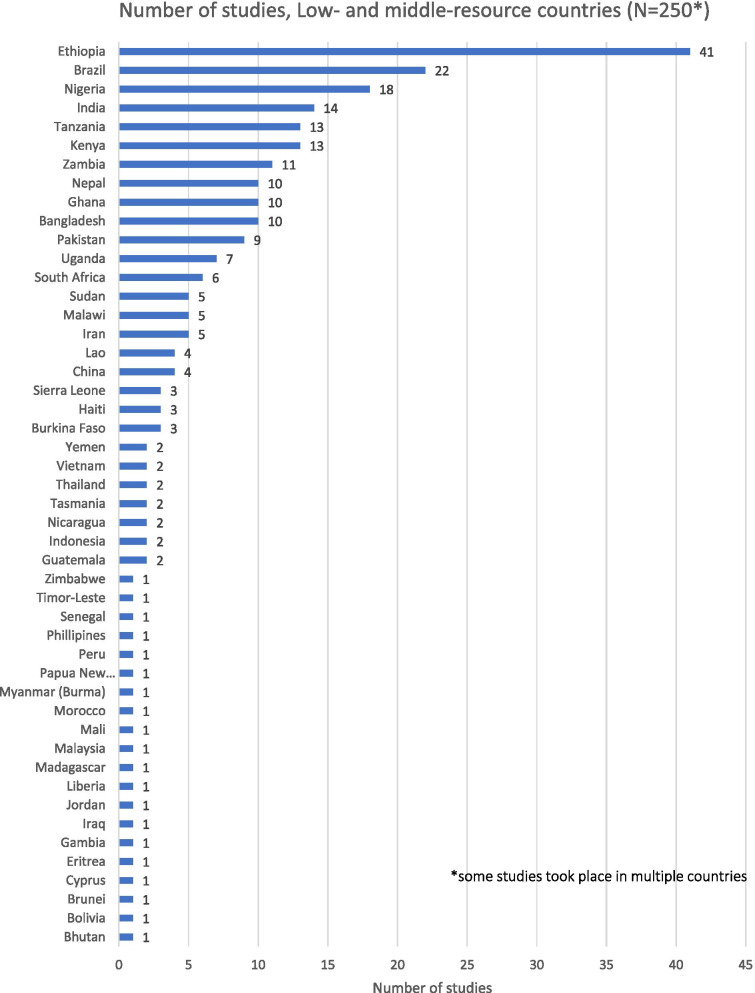
Low- and middle resource countries conducting stakeholder research

A slightly higher proportion of the included studies took place in low- and middle-resource countries (LMRCs): a total of 250 studies eliciting the views of stakeholders about home birth across 48 countries. This is shown in Fig. 5.

**Fig. 6:**
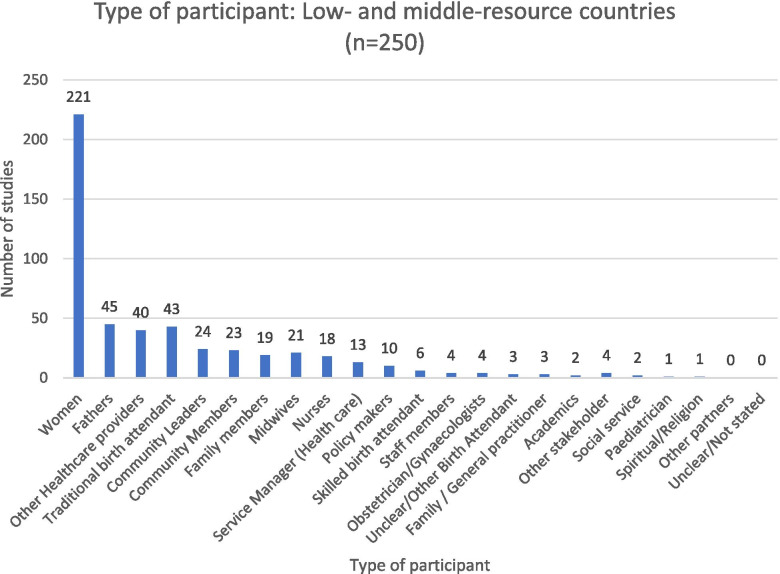
Low- and middle-resource countries: type of participant

Studies of stakeholder perspectives of home birth were conducted in multiple LMRCs, as illustrated in Fig. 6.

The largest body of research in this group has been conducted in Ethiopia (41 studies, 16%). A modest number of studies have been conducted in other countries with high rates of maternal mortality including Brazil (9%), Nigeria (7%), India (6%), Tanzania and Kenya (5%), and Zambia, Nepal, Ghana, and Bangladesh (4% each). Countries with the highest maternal mortality rates (over 1000 per 100,000 live births) as defined by the WHO, UNICEF, the UN Population Fund, and the World Bank [72] were partially represented: three studies were located in Sierra Leone and four studies in Sudan (including south Sudan). However, no research on stakeholder perspectives of home birth could be located originating from Chad. The distribution of included studies across countries with high, very high, and extremely high maternal mortality is further illustrated in Table 1.

**Table 1:** Number of studies conducted in countries of high, very high or extremely high maternal mortality

**Country**	**Number of studies**
**Extremely high maternal mortality** (> 1000 maternal deaths per 100,000 (100K) live births)	
Chad	0
Sierra Leone	3
South Sudan/Sudan	5
**Very high maternal mortality** (500–999 maternal deaths per 100,000 (100K) live births)	
Afghanistan	0
Cameroon	0
Central Republic of Africa	0
Côte d’Ivoire	0
Guinea	0
Guinea-Bissau	0
Mauritania	0
Niger	0
Somalia	0
Liberia	1
Mali	1
Tanzania	13
Nigeria	18
**High maternal mortality** (300–499 maternal deaths per 100,000 (100K) live births)	
Benin	0
Congo	0
Democratic Republic of the Congo	0
Equatorial Guinea	0
Togo	0
Eritrea	1
Madagascar	1
Zimbabwe	1
Senegal (including Gambia)	2
Burkina Faso	3
Malawi	5
Uganda	6
Ghana	10
Kenya	13
Ethiopia	41

As defined in WHO, UNICEF, UN Population Fund, World Bank, *Trends in Maternal Mortality: 2000 to 2017* WHO, Geneva, 2019

#### Populations studied in low- and middle-resource countries

As in studies conducted in HRCs, the perspectives of women experiencing home birth in LMRCs were most often sought (221 of 250 LMRC studies, 88%). This is shown in Fig. 6.

Many other types of stakeholders participated in home birth research studies across LMRCs. Fathers perspectives were explored in 45 studies (18%), as were family members (8%). A diversity of healthcare providers perspectives were also studied, including outreach workers, health extension workers, and unspecified health workers (17%). The experiences of traditional birth attendants were reported in similar proportions (17%), followed by midwives (8%), nurses (7%), and skilled birth attendants (2%). The experiences of service managers (5%) and staff members and obstetricians (2% each), family practitioners (three studies), and pediatricians (one study) were less often reported.

A modest number of studies within LMRCs included the perspectives or community leaders (10%), followed by community members (9%), and policy makers (4%). Perspectives of academics or social service personnel (two studies each) and religious leaders (one study) were less often stated.

#### Home birth issues studied in low- and middle-resource countries

**Fig. 7:**
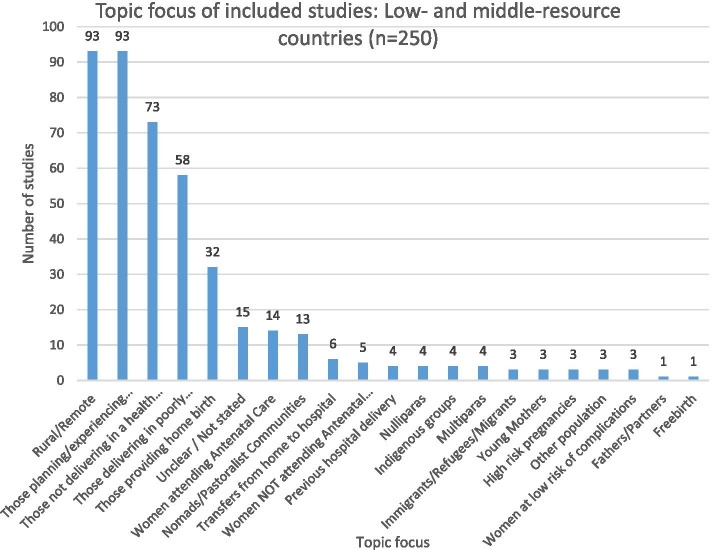
Topic focus of included studies: low- and middle-resource countries

A wide range of topics were studied across LMRCs, as shown in Fig. 7.

Within LMRCs, participants’ perspectives shaped by geographic and economic determinants were evidenced, with studies of those experiencing rural or remote home birth (93 studies, 29% of 206 LMRC studies), in poorly resourced areas (20%), nomadic or pastoralist communities (5%) studied.

Almost one third of the studies in LMRCs sought to understand home birth from the perspective of those people experiencing home birth (93 of the 250 studies conducted in LMRCs, 29%). The focus of those providing home birth services was examined in 32 studies (9%).

Many of the studies conducted in LMRCs sought to understand why home birth took place despite an emphasis on promoting health facility delivery (26%). Other related studies examined perspectives of women who experienced home birth despite receiving antenatal care (6%), or not utilizing antenatal care (2%). The views of those women experiencing previous hospital delivery were reported in four studies (2%). Another six studies (2%) examined the views of those experiencing transfer from home to hospital.

Specific populations at risk of poor outcomes were less often studied, including first time mothers, multiparas, and Indigenous groups (four studies each), refugees and migrants, and those with high-risk pregnancies (three studies each). Studies originating in LMRCs with a specific focus on the perspectives of fathers only were rarely reported (one study).

## Discussion

### Stakeholder-focused research

It has long been considered important that health policy and practice decisions should be informed by the perspectives of those who plan, provide and are affected by them [73]. The research literature on women’s perspectives of home birth in high-resource countries including the UK and Brazil have been systematically reviewed [31, 39, 41]. However, these syntheses are in need of updating. The large number of recent research from HRCs on women’s perspectives of home birth could address this need. While current efforts are underway to review the research on women’s perspectives of home birth across all high-resource countries [34, 35], consideration should be given to the differences between countries in terms of the nature and quality of health care provision, including the integration of home birth services [9]. There is also a need to understand women’s experiences of home birth in LMRCs. Research with women in LMRCs comprised the largest body of evidence located in our scoping review. This evidence has been systematically reviewed and synthesized for home birth in Ethiopia [74], Ghana [75], and Brazil [76]. Studies examining factors influencing and perspectives of home birth in Ethiopia and Ghana note similar barriers to hospital delivery due to access [74, 75]. Others note that planned home birth in Brazil is most often utilized by women of higher socioeconomic status [77], but there is scarce and poor quality research regarding midwives’ role in Brazilian home birth [76]. Examination of these perspectives of women and birth attendants, both within and across countries with the highest maternal mortality, could highlight areas where health systems could be strengthened. While a large body of research was located examining nulliparous women’s views of home birth in high-income countries, this has yet to be integrated into a qualitative evidence synthesis. Similarly, the primary evidence on multiparous women’s experiences of home birth in HRCs has accumulated and could usefully update and inform previous research syntheses of this population [30].

In HRCs, a body of research was located on fathers’ and same-sex partners’ experiences of home birth. A synthesis of the existing literature on these partners’ perspectives of home birth in high resource countries is an important area of need, as this appears to be a new area of birth research. In LMRCs, it has been suggested that fathers and other family members influence women’s health care decisions, including place of birth [78–82]. Our scoping review identified several studies eliciting the views of fathers and other family members in LMRCs about home birth, which could be synthesized usefully. Integration of this evidence could further identify the ways in which family members influence the decision on women’s place of birth in LMRCs [83, 84].

A substantial number of studies examining LMRC community members’ views of home birth were also located. This could further supplement existing evidence syntheses of community interventions to prevent maternal and newborn mortality [85, 86]. In addition, a small body of literature was located examining the perspectives of community leaders and policy makers in LMRCs regarding home birth. Synthesizing this literature would provide an understanding of the health system factors that must be leveraged in order to integrate midwifery and skilled birth attendant home birth services into LMRC health system infrastructure.

Health professionals’ perspectives of home birth also hold the potential to inform maternity policy and practice; however, only one previous systematic review was located which examined midwives’ discussions of place of birth options with women in HRCs [40]. This scoping review located multiple primary studies that assessed the views of midwives, medical staff, and other health care providers in high-resource countries. Further synthesis of this literature would add to the existing evidence and could expand our understanding of the views of other health care providers in high-resource settings. In LMRCs, one recent systematic review examined the perspectives of midwives and skilled birth attendants concerning home birth [42]. This evidence could be enhanced and updated by synthesizing the evidence located in our scoping review which identified research with community health workers, health extension workers, lady health workers, and service managers and staff members. By understanding these perspectives, birth services may be configured to address key stakeholders’ concerns.

### Issue-focused research

Several topics or issues of interest also emerged from the analysis of HRC research on stakeholder views of home birth. This included new primary research on people’s perspectives of freebirth or unassisted birth, where women choose to birth without a trained professional present, even where there is access to medical facilities. Previous syntheses of this research suggest that women make this choice, for reasons of autonomy, choice, and control over their own bodies, due to midwives and current maternity services [32, 87]. This body of literature is currently being further synthesized in order to supplement previous research in this area [88, 89].

Similarly, our scoping review identified multiple research studies of stakeholder perspectives on home birth in high-risk pregnancy, in those who had a previous hospital delivery and among women experiencing transfer from home to hospital. Integration of this body of research conducted in high-resource settings could broaden our understanding of home birth with reference to safety and risk. For example, this could update an existing evidence synthesis examining perspectives of transfer from home to hospital [33]. This focus was also seen in LMRC-based studies. Several studies focused on stakeholder perspectives of transfer from home to hospital, their experiences of home birth in high-risk pregnancy, and the experiences of those who give birth at home despite access to antenatal care and/or hospital facilities. A synthesis of these topics would update and extend prior outdated work in this area [90].

Several studies originating in LMRCs also examined stakeholder perspectives of home birth where the population is rural, remote, or has poor access to health services. It has been noted that poor geographic access to hospital facilities in LMRCs impedes health care utilization [91]. Stakeholder research of rural and remote access and home birth in HRC populations has been previously examined [36, 37] but has yet to be synthesized for populations living in LMRCs. A synthesis of this literature could further inform understanding of the factors influencing where women give birth and how health systems infrastructure could better support childbearing women in rural and remote locations.

### Gaps in primary stakeholder research on home birth

This scoping review of stakeholder research about home birth also identified several gaps in primary research. For example, in high-resource countries, very little research was identified which sought the views of same-sex childbearing partners, or other family members, such as grandparents. Given the influence of family members on women’s birth decisions [78, 81, 82], this constitutes an important gap in the evidence. Perspectives of those experiencing home birth in high-resource rural and remote locations is also lacking. This evidence could help to inform maternity service provision and personal decisions. While several studies of Indigenous perspectives of home birth exist in both high- and low- and middle-resource countries [92, 93], more recent research in this area could reflect recent changes to service provision in some high-resource countries [54, 94].

The views of specific populations concerning home birth also bear further examination. These include the perspectives of stakeholders in countries of higher maternal mortality, including countries in sub-Saharan Africa and Southeast Asia [20].

Research on the perspectives of first-time mothers and multiparas, whose experiences may differ from one another, could also suggest different avenues of health system support.

In addition, primary research seeking young mothers’ perspectives of home birth was rarely located. Given that the highest rate of maternal mortality in LMRCs occurs in women aged 10 to 14 years, this is an important area of future research [20].

Finally, primary stakeholder research on home birth among specific and potentially more vulnerable populations is also lacking in both HRCs and LMRCs, including Indigenous populations, immigrants, refugees, and migrants. Displaced populations are at higher risk of maternal mortality due to the associated conflict and humanitarian crises [72]. This suggests that this may also be a very useful area of future primary research inquiry to influence maternal service provision in these fragile states [72].

### Strengths and limitations of the review

The main strength of this systematic scoping review lies in its broad search for relevant literature and consistent methods of screening, coding and analysis. To our knowledge, this is the first representation of the landscape of qualitative literature worldwide focused specifically on stakeholders’ experience and perspectives of home birth. As such, it provides a valuable resource for researchers, members of the public and decision-makers for informing personal and policy decisions related to home birth and identifies areas for future research.

The results of this review are limited by the depth of analysis possible using scoping review methods, a challenge noted by others [95, 96]. The breadth of research and resources available limit the amount of data extraction and analysis possible [63]. However, the intent of this scoping review was to inform subsequent qualitative evidence syntheses. To mitigate this, data extraction was designed in consultation with an advisory group of stakeholders to capture key characteristics of the studies, which will inform future collaborative research decisions.

## Conclusions

Our systematic scoping review identified a large body of research literature that privileged the experiences and perspectives of a wide range of key stakeholders about home birth in both high and low and middle income countries.

Groups of primary research focused on different topics and populations within HRCs and LMRCs could be usefully synthesized to inform personal practice and policy decisions. However, these research syntheses would be best informed by collaboration between researchers and childbearing women and their support people, clinicians, professional organizations, research funders, and clinical and policy decision-makers. These stakeholders can better inform personal and policy decisions by working together to collectively identify emerging issues, priorities, and the associated research questions [73, 97, 98]. Important gaps in primary research should also be addressed with respect to perspectives of family members in HRCs, stakeholders in countries with high maternal mortality, young mothers and Indigenous populations, immigrants, migrants, and refugees.

## Data Availability

The datasets generated and analyzed during the current study are not publicly available due to their generation and maintenance on third party software, but are available from the corresponding author on reasonable request.

## Appendix

### Search strategies and outputs

#### Home birth scoping review: search strategy

**Initial Search Feb 2020**

**OVID MEDLINE** [1720 results]

Ovid Medline Syntax Legend:

adj2 = within 2 words in either direction (“delivery in the home”, “home delivery”, “home when delivering” etc.)

* Asterisk brings back word endings (deliver* = deliver, delivers, delivery, deliveries)

* Asterisk in front of a subject heading = focus; this subject heading is a major one

.tw. = text word (searching the title and abstract in Ovid Medline)

/ = subject headings

1. (Home adj2 (childbirth* or child birth* or birth* or waterbirth*)).tw.
2. homebirth*.tw.
3. (outside adj3 hospital).tw.
4. (childbirth* or child birth* or birth* or waterbirth*).tw.
5. 3 and 4
6. (home adj2 deliver*).tw.
7. (childbirth* or child birth* or birth* or waterbirth*).tw.
8. 6 and 7
9. *Home Childbirth/
10. 1 or 2 or 5 or 8 or 9
11. Limit 1 Jan 2010 – 31 Dec 2019

**EBSCOHost CINAHL** [1775 results]

CINAHL Syntax Legend:

N2 = within 2 words in either direction (“delivery in the home”, “home delivery”, “home when delivering” etc.)

W3 = within 3 words in that exact order (“outside of the hospital”)

* Asterisk brings back word endings (deliver* = deliver, delivers, delivery, deliveries)

“” = quotes around phrases (i.e., “child birth*”)

MM = focus; this subject heading is a major one (MM “Home Childbirth”)

TI = title

AB = abstract

- Equivalent to text word .tw. in Ovid Medline which looks at title and abstract only

1. TI ( home N2 (childbirth* or “child birth*” or birth* or waterbirth*) ) OR AB ( home N2 (childbirth* or “child birth*” or birth* or waterbirth*) )
2. TI homebirth* OR AB homebirth*
3. TI outside W3 hospital OR AB outside W3 hospital
4. TI ( childbirth* or “child birth*” or birth* or waterbirth* ) OR AB ( childbirth* or “child birth*” or birth* or waterbirth* )
5. S3 AND S4
6. TI home N2 deliver* OR AB home N2 deliver*
7. TI ( childbirth* or “child birth*” or birth* or waterbirth* ) OR AB ( childbirth* or “child birth*” or birth* or waterbirth* )
8. S6 AND S7
9. (MM “Home Childbirth”)
10. 1 OR 2 OR 5 OR 8 OR 9
11. Limit 1 Jan 2010 – 31 Dec 2019

**OVID Cochrane Library** [312 results]

1. (Home adj2 (childbirth* or child birth* or birth* or waterbirth*)).tw.
2. homebirth*.tw.
3. (outside adj3 hospital).tw.
4. (childbirth* or child birth* or birth* or waterbirth*).tw.
5. 3 and 4
6. (home adj2 deliver*).tw.
7. (childbirth* or child birth* or birth* or waterbirth*).tw.
8. 6 and 7
9. *Home Childbirth/
10. 1 or 2 or 5 or 8 or 9
11. Limit 1 Jan 2010 – 31 Dec 2019

**ProQuest ASSIA** [143 results]

Via UCL Library

1. (ab,ti)home NEAR/2 childbirth*
2. (ab,ti)home NEAR/2 child birth*
3. (ab,ti)home NEAR/2 birth*
4. 1 OR 2 OR 3
5. (ab,ti)homebirth*
6. (ab,ti)outside NEAR/3 hospital*
7. ((ab,ti) childbirth*) OR ab,ti(child birth*) OR ab,ti(birth*) OR ab,ti(waterbirth*)
8. 6 AND 7
9. (ab,ti)home NEAR/2 deliver*
10. ((ab,ti) childbirth*) OR ab,ti(child birth*) OR ab,ti(birth*) OR ab,ti(waterbirth*)
11. 9 AND 10
12. MAINSUBJECT.EXACT(“Home birth”)
13. 4 OR 5 OR 8 OR 11 OR 12

**OVID EMBASE** [1831 results]

Via UCL Library

1. (Home adj2 (childbirth$ or child birth$ or birth$ or waterbirth$)).tw.
2. homebirth*.tw.
3. (outside adj3 hospital).tw.
4. (childbirth* or child birth* or birth* or waterbirth*).tw.
5. 3 and 4
6. (home adj2 deliver*).tw.
7. (childbirth* or child birth* or birth* or waterbirth*).tw.
8. 6 and 7
9. *Home Childbirth/
10. 1 or 2 or 5 or 8 or 9
11. Limit 1 Jan 2010 – 31 Dec 2019

**HBSR Update Search July 2020**

**OVID MEDLINE** [132 results]

1. (Home adj2 (childbirth* or child birth* or birth* or waterbirth*)).tw.
2. homebirth*.tw.
3. (outside adj3 hospital).tw.
4. (childbirth* or child birth* or birth* or waterbirth*).tw.
5. 3 and 4
6. (home adj2 deliver*).tw.
7. (childbirth* or child birth* or birth* or waterbirth*).tw.
8. 6 and 7
9. *Home Childbirth/
10. 1 or 2 or 5 or 8 or 9
11. Limit 1 Jan 2020 – 30 June 2020

**EBSCOHost CINAHL** [191 results]

1. TI (home N2 (childbirth* or “child birth*” or birth* or waterbirth*) ) OR AB ( home N2 (childbirth* or “child birth*” or birth* or waterbirth*) )
2. TI homebirth* OR AB homebirth*
3. TI outside W3 hospital OR AB outside W3 hospital
4. TI ( childbirth* or “child birth*” or birth* or waterbirth* ) OR AB ( childbirth* or “child birth*” or birth* or waterbirth* )
5. S3 AND S4
6. TI home N2 deliver* OR AB home N2 deliver*
7. TI ( childbirth* or “child birth*” or birth* or waterbirth* ) OR AB ( childbirth* or “child birth*” or birth* or waterbirth* )
8. S6 AND S7
9. (MM “Home Childbirth”)
10. 1 OR 2 OR 5 OR 8 OR 9
11. Limit 1 Jan 2020 – 30 June 2020

**OVID Cochrane Library** [22 results]

1. (Home adj2 (childbirth* or child birth* or birth* or waterbirth*)).tw.
2. homebirth*.tw.
3. (outside adj3 hospital).tw.
4. (childbirth* or child birth* or birth* or waterbirth*).tw.
5. 3 and 4
6. (home adj2 deliver*).tw.
7. (childbirth* or child birth* or birth* or waterbirth*).tw.
8. 6 and 7
9. *Home Childbirth/
10. 1 or 2 or 5 or 8 or 9
11. Limit 1 Jan 2020 – 30 June 2020

**ProQuest ASSIA** [84 results]

1. (ab,ti)home NEAR/2 childbirth*
2. (ab,ti)home NEAR/2 child birth*
3. (ab,ti)home NEAR/2 birth*
4. 1 OR 2 OR 3
5. (ab,ti) homebirth*
6. (ab,ti)outside NEAR/3 hospital*
7. ((ab,ti) childbirth*) OR ab,ti(child birth*) OR ab,ti(birth*) OR ab,ti(waterbirth*)
8. 6 AND 7
9. (ab,ti)home NEAR/2 deliver*
10. ((ab,ti) childbirth*) OR ab,ti(child birth*) OR ab,ti(birth*) OR ab,ti(waterbirth*)
11. 9 AND 10
12. MAINSUBJECT.EXACT(“Home birth”)
13. 4 OR 5 OR 8 OR 11 OR 12
14. Limit 1 Jan 2020 – 30 June 2020

**EMBASE** [109 results]

1. (Home adj2 (childbirth$ or child birth$ or birth$ or waterbirth$)).tw.
2. homebirth*.tw.
3. (outside adj3 hospital).tw.
4. (childbirth* or child birth* or birth* or waterbirth*).tw.
5. 3 and 4
6. (home adj2 deliver*).tw.
7. (childbirth* or child birth* or birth* or waterbirth*).tw.
8. 6 and 7
9. *Home Childbirth/
10. 1 or 2 or 5 or 8 or 9
11. Limit 1 Jan 2020 – 30 June 2020

**PROQUEST Dissertations & Theses** (14 hits)

1. ti(Home birth) OR ab(Home birth)
2. ti(Home childbirth) OR ab(Home childbirth)
3. ti(Home delivery) OR ab(Home delivery)
4. ti(Homebirth) OR ab(Homebirth)
5. 1 OR 2 OR 3 OR 4
6. Limit 1 Jan 2020 – 30 June 2020

**HBSR Update Search April 2021**

**OVID MEDLINE** [123 results]

1. (Home adj2 (childbirth* or child birth* or birth* or waterbirth*)).tw.
2. homebirth*.tw.
3. (outside adj3 hospital).tw.
4. (childbirth* or child birth* or birth* or waterbirth*).tw.
5. 3 and 4
6. (home adj2 deliver*).tw.
7. (childbirth* or child birth* or birth* or waterbirth*).tw.
8. 6 and 7
9. *Home Childbirth/
10. 1 or 2 or 5 or 8 or 9
11. Limit 1 Jul 2020 – 30 Apr 2021

**EBSCOHost CINAHL** [224 results]

1. TI (home N2 (childbirth* or “child birth*” or birth* or waterbirth*) ) OR AB ( home N2 (childbirth* or “child birth*” or birth* or waterbirth*) )
2. TI homebirth* OR AB homebirth*
3. TI outside W3 hospital OR AB outside W3 hospital
4. TI ( childbirth* or “child birth*” or birth* or waterbirth* ) OR AB ( childbirth* or “child birth*” or birth* or waterbirth* )
5. S3 AND S4
6. TI home N2 deliver* OR AB home N2 deliver*
7. TI ( childbirth* or “child birth*” or birth* or waterbirth* ) OR AB ( childbirth* or “child birth*” or birth* or waterbirth* )
8. S6 AND S7
9. (MM “Home Childbirth”)
10. 1 OR 2 OR 5 OR 8 OR 9
11. Limit 1 Jul 2020 – 30 Apr 2021

**Cochrane Library** [49 results]

1. (Home adj2 (childbirth* or child birth* or birth* or waterbirth*)).tw.
2. homebirth*.tw.
3. (outside adj3 hospital).tw.
4. (childbirth* or child birth* or birth* or waterbirth*).tw.
5. 3 and 4
6. (home adj2 deliver*).tw.
7. (childbirth* or child birth* or birth* or waterbirth*).tw.
8. 6 and 7
9. *Home Childbirth/
10. 1 or 2 or 5 or 8 or 9
11. Limit 1 Jul 2020 – 30 Apr 2021

**ProQuest ASSIA** [16 results]

1. (ab,ti)home NEAR/2 childbirth*
2. (ab,ti)home NEAR/2 child birth*
3. (ab,ti)home NEAR/2 birth*
4. 1 OR 2 OR 3
5. (ab,ti) homebirth*
6. (ab,ti)outside NEAR/3 hospital*
7. ((ab,ti) childbirth*) OR ab,ti(child birth*) OR ab,ti(birth*) OR ab,ti(waterbirth*)
8. 6 AND 7
9. (ab,ti)home NEAR/2 deliver*
10. ((ab,ti) childbirth*) OR ab,ti(child birth*) OR ab,ti(birth*) OR ab,ti(waterbirth*)
11. 9 AND 10
12. MAINSUBJECT.EXACT(“Home birth”)
13. 4 OR 5 OR 8 OR 11 OR 12
14. Limit 1 Jul 2020 – 30 Apr 2021

**EMBASE** [310 results]

1. (Home adj2 (childbirth$ or child birth$ or birth$ or waterbirth$)).tw.
2. homebirth*.tw.
3. (outside adj3 hospital).tw.
4. (childbirth* or child birth* or birth* or waterbirth*).tw.
5. 3 and 4
6. (home adj2 deliver*).tw.
7. (childbirth* or child birth* or birth* or waterbirth*).tw.
8. 6 and 7
9. *Home Childbirth/
10. 1 or 2 or 5 or 8 or 9
11. Limit 1 Jul 2020 – 30 Apr 2021

**PROQUEST Dissertations & Theses** (14 hits)

1. Home birth
2. Home childbirth
3. Home delivery
4. Homebirth
5. 1 OR 2 OR 3 OR 4
6. Limit 1 July 2020 – 30 April 2021

**Websites/Key journals**

WHO “Home Birth” in Publications or Articles since 2020 – None

ICM – “home birth” – None

CJMRP – 0 new since 2019

Br J Midw – 1 hits

Midwifery – 15 hits

## Abbreviations

HRCs: High-resource countries
LMRCs: Low- and middle-resource countries
